# Computational Analysis of Chromophore‐Controlled Photoactivation in Monofunctional Platinum(II)–BODIPY Conjugates for Dual Chemo‐Photodynamic Therapy

**DOI:** 10.1002/cbic.70422

**Published:** 2026-06-11

**Authors:** Fortuna Ponte, Gloria Mazzone

**Affiliations:** ^1^ Department of Chemistry and Chemical Technologies University of Calabria Rende Italy

**Keywords:** BODIPY, metal‐chromophore conjugates, PDT, Pt(II) complexes, TD‐DFT

## Abstract

Platinum(II)‐based photoactivatable anticancer agents that combine chemotherapy and photodynamic therapy (PDT) represent a promising strategy to overcome resistance and systemic toxicity of classical platinum drugs. Herein, we report a density functional theory (DFT) and time‐dependent DFT investigation of dipicolylamine (dpa)‐based monofunctional Pt(II) complexes conjugated to boron dipyrromethene (BODIPY) chromophores. Starting from two experimentally characterized systems, we designed new Pt–BODIPY architectures aimed at shifting light absorption into the therapeutic window (600–850 nm) while preserving platinum reactivity toward DNA. The results demonstrate that extension of π‐conjugation and appropriate linker engineering effectively redshift the absorption maximum up to 661 nm without compromising capability of singlet oxygen generation. All conjugates exhibit triplet energies (1.03–1.57 eV) sufficient to sensitize molecular oxygen and calculated intersystem crossing (ISC) rates typical of chromophore‐dominated systems, with limited spin–orbit coupling (SOC) enhancement from the Pt center. Mechanistic analysis of aquation and guanine coordination shows activation barriers and thermodynamics comparable to conventional Pt(II) drugs, indicating that chromophore conjugation does not hinder DNA platination. Overall, this study provides structure–property relationships and rational guidelines for the development of dual‐action Pt–BODIPY chemo‐photodynamic agents.

## Graphical abstract

BODIPY‐decorated monofunctional Pt(II) complexes were computationally designed as dual chemo‐photodynamic agents. π‐conjugation engineering shifts absorption into the therapeutic window (up to 661 nm) while preserving singlet oxygen generation and DNA platination capability. The study reveals how chromophore design governs photoactivity without compromising platinum reactivity.

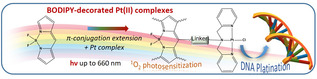

## 1 Introduction

Since the discovery of cisplatin antineoplastic activity, platinum(II)‐based anticancer drugs play a central role in cancer chemotherapy for the treatment of a wide variety of tumors [1, 2, 3, 4, 5, 6, 7, 8]. Their mechanism of action has been extensively elucidated in its essential aspects [9, 10]. Upon activation by hydrolysis, cisplatin‐like compounds exert their cytotoxic effects through covalent binding to nuclear DNA, forming DNA cross‐links. This interaction induces significant DNA distortion, inhibits transcription and replication processes, and finally results in cellular apoptosis.

Despite the widespread use of cisplatin and Pt(II) derivatives, their therapeutic application is hampered by dose‐dependent toxicity, poor tumor selectivity and restricted access to nuclear DNA. In particular, lysosomal sequestration of platinum complexes has increasingly been recognized as a major barrier to therapeutic efficacy, as it prevents nuclear accumulation and contributes to resistance mechanisms [11, 12, 13]. While this phenomenon is typically considered detrimental, it also offers an opportunity to temporarily silence platinum drugs as prodrugs and activate them selectively within tumor cells [14, 15, 16].

These drawbacks have stimulated considerable efforts to design new platinum‐based compounds that work via alternative mechanisms of action and ideally display enhanced therapeutic efficacy. Among the proposed strategies to overcome the side effects of classical bifunctional platinum drugs, monofunctional platinum(II) complexes emerged like promising nonclassical alternatives [17, 18, 19, 20]. Unlike cisplatin, which forms intrastrand and interstrand DNA cross‐links via two labile ligands, monofunctional platinum complexes, possessing only one leaving group, can form only a single covalent bond with DNA; thereby reducing transcription‐coupled DNA repair. Apoptosis can consequently be induced through alternative mechanisms of action, including additional noncovalent interactions and steric effects [21].

Moreover, recent advances have demonstrated that photoactivatable platinum complexes can exploit lysosomal trapping behavior to achieve spatial and temporal control over drug activation. By integrating a photosensitizing chromophore (PS) with a monofunctional Pt(II) center, such systems can remain biologically inert in the dark while undergoing light‐triggered cytotoxic species generation upon irradiation. In particular, the localized oxidative stress generated by the photoactivated chromophore can compromise lysosomal membrane integrity, promoting lysosomal membrane permeabilization and the subsequent release of the platinum complex into the cytosol and nucleus [14, 22]. This mechanism opens new perspectives in cancer treatment through the combination of classical Pt chemotherapy with photodynamic therapy (PDT) [23].

PDT is a clinically approved light‐assisted therapeutic treatment that exhibits low systemic toxicity. It is already employed to treat a variety of tumors by generating oxygen‐based cytotoxic species (ROSs) that induce oxidative damage to cellular components and ultimately trigger cell death. PDT relies on the administration of a photosensitizer that, upon irradiation with light of an appropriate wavelength, is excited and subsequently undergoes intersystem crossing (ISC) to a triplet excited state. This long‐lived triplet state can efficiently transfer energy to molecular oxygen, resulting in the formation of highly cytotoxic singlet oxygen (^1^O_2_) through a type II photochemical pathway, or produce other reactive radical species via a type I photoreaction. The efficacy of PDT strongly depends on the photophysical properties of the photosensitizer, particularly its ability to absorb light within the 600–850 nm therapeutic window, where tissue penetration is maximized.

In this context, Pt(II)‐chromophore conjugates represent particularly attractive systems, as incorporation of a Pt center in principle may enhance spin–orbit coupling (SOC) through relativistic heavy‐atom effects. However, the extent of this enhancement strongly depends on the degree of electronic communication between the metal fragment and the chromophore‐centered excited states [24, 25, 26]. Simultaneously, photoinduced ROS can deplete intracellular glutathione levels, stabilizing further the platinum species and enhancing their DNA‐binding capability. Overall, this combination overcomes limitations associated with conventional anticancer drugs while improving intracellular drug distribution and therapeutic efficacy [23, 27, 28].

Among the photosensitizers potentially linkable to Pt(II) complexes, boron dipyrromethene (BDP) dyes have been proposed as promising chromophores thanks to their very valuable chemical‐physical properties, such as high thermodynamic stability, high molar extinction coefficients, and tunable absorption/emission characteristics [29, 30, 31, 32]. In this framework, two investigated conjugates based on dipicolylamine (dpa) monofunctional platinum(II) systems have been shown to exert multiple cytotoxic activities once activated by light [22, 33].

**SCHEME 1:**
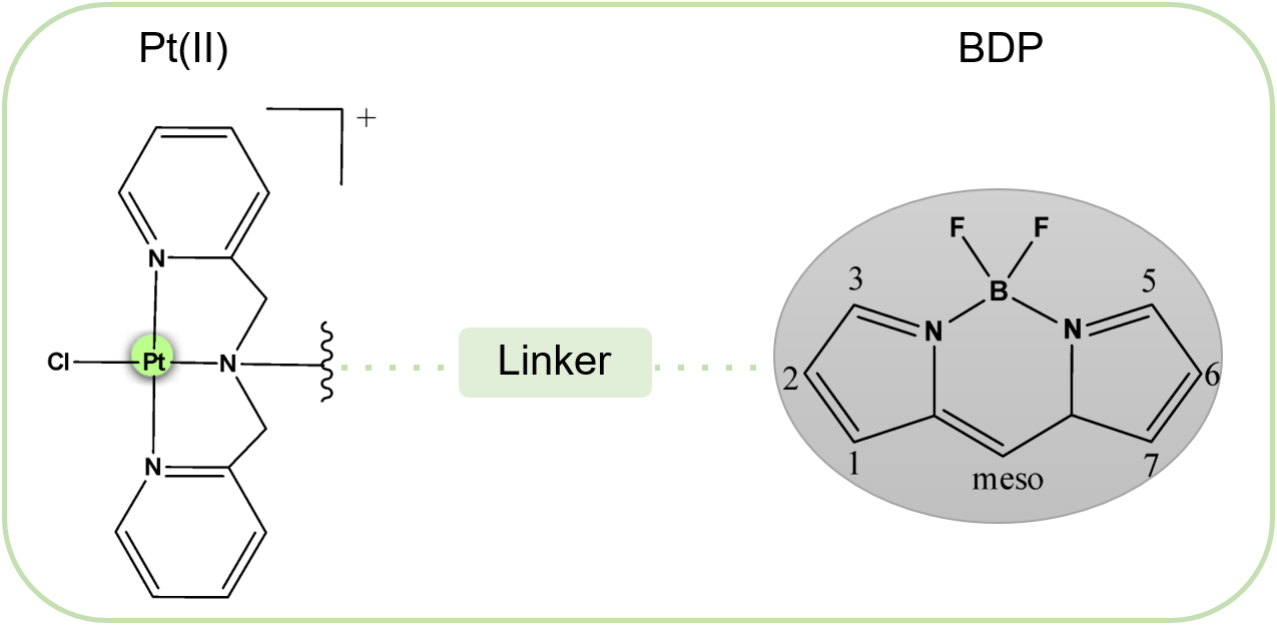
Molecular architecture of the investigated Pt‐BDP conjugates.

The present computational work aims to contribute to the rational design of novel BDP‐based photosensitizers incorporating dpa‐based monofunctional platinum(II) systems, thereby developing photocytotoxic systems in which the BDP moiety acts as a type‐II photosensitizer. Starting from the previously reported experimental studies on dpa‐based platinum‐BDP conjugates [22, 33], we propose new molecular architectures with the aim to shift the maximum absorption within the therapeutic window (600–850 nm) while maintaining the cytotoxicity of the platinum center. A schematic representation of the main components of the investigated conjugates is shown in Scheme 1, highlighting the three essential elements of the molecular architecture: a) dipicolylamine‐based (dpa) monofunctional platinum(II) complex (Pt), b) the motif linking the complex and the chromophore (linker), and c) the chromophore (BDP). Within this framework, the present study focuses on three aspects: (i) investigating how structural modifications of the BODIPY scaffold influence the photophysical properties of the conjugates; (ii) evaluating the accessibility of triplet excited states relevant for PDT applications; and (iii) assessing whether the monofunctional Pt(II) fragment retains the ability to undergo aquation and coordinate nucleobases in a manner analogous to cisplatin‐like compounds.

To this end, the main photophysical properties together with the potential DNA binding of the complexes have been computationally explored by means of density functional theory (DFT) and its time‐dependent formulation (TDDFT). Overall, this study provides theoretical insights into the structure–property relationships that modulate the photophysical and photochemical behavior of dpa‐based monofunctional Pt(II) complexes and offers guidelines for developing new dual‐action chemo‐PDT agents optimized for activation within the therapeutic window.

## 2 Results and Discussion

**SCHEME 2:**
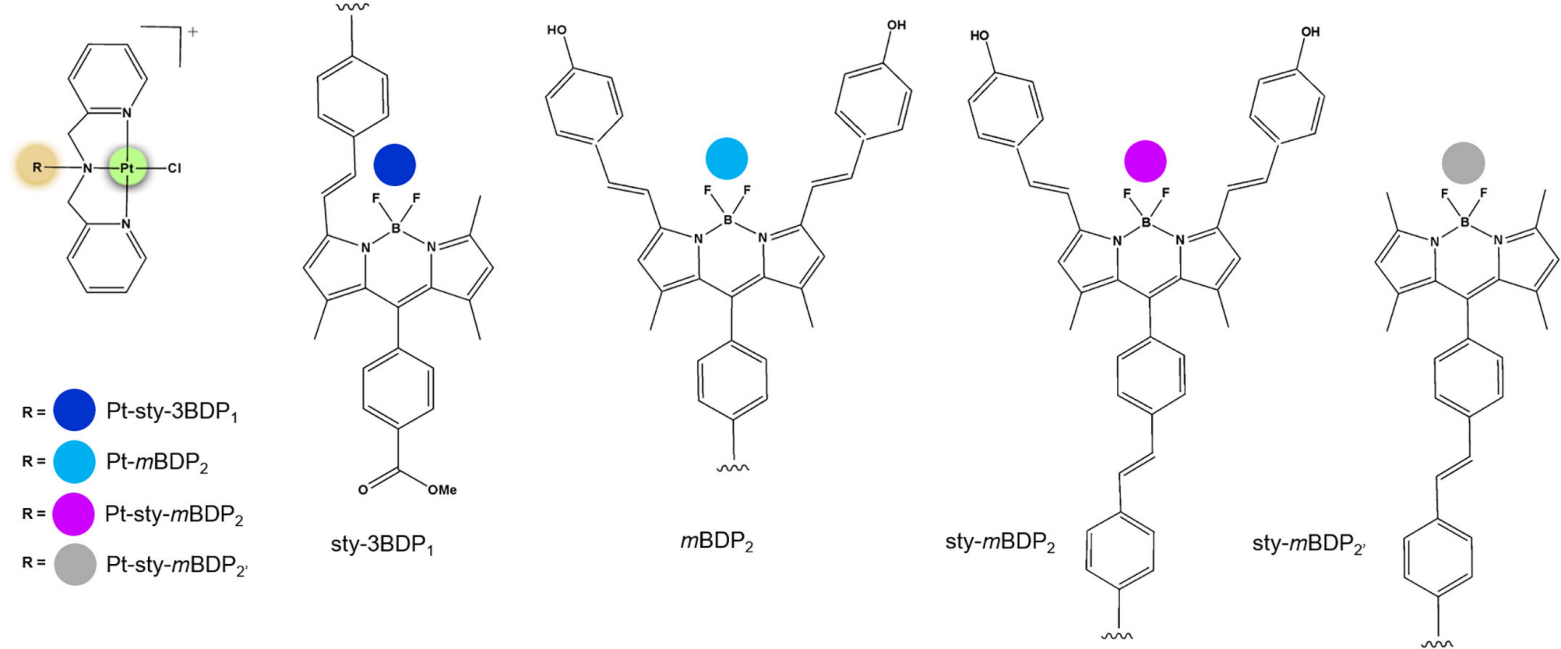
Schematic representation of the investigated complexes, Pt‐sty‐3BDP_1_, Pt‐*m*BDP_2_, Pt‐sty‐*m*BDP_2_, and Pt‐sty‐*m*BDP_2_.

A schematic overview of all the investigated complexes is shown in Scheme 2. The naming convention adopted for the conjugates highlights the three fundamental structural components: the dpa‐based Pt(II) fragment, the linker bridging the metal center and the chromophore, and the BDP scaffold, including the specific attachment site (3‐ or *meso*‐position) of the platinum‐containing unit.

The impact of the Pt(II) fragment on the photochemical behavior of the BDP chromophore was investigated by analyzing the electronic absorption spectra and calculating the ISC rate constants (k_ISC_). In parallel, the influence of the BDP photosensitizer on platinum activation was examined through a mechanistic study of the hydrolysis process, specifically the substitution of the chlorido leaving group by a water molecule, which represents the first essential step preceding drug–DNA interaction involving cisplatin‐like Pt(II) compounds. Subsequently, the interaction between the aquated Pt conjugates and the purine nucleobase guanine was evaluated to model the platination step. For such a class of drugs, this process, corresponding to coordination at the *N*7 position of guanine, leads to distortion of the DNA double helix, and ultimately inhibits replication and transcription.

### 2.1 Structural, Electronic, and Optical Properties

Complexes Pt‐sty‐3BDP_1_ and Pt‐*m*BDP_2_ are the conjugates that were previously synthesized and evaluated in vitro [22, 33]. These compounds served as reference systems for the rational, computationally driven design of two novel monofunctional platinum‐BDP conjugates aimed at achieving improved photosensitizing properties, named Pt‐sty‐*m*BDP_2_ and Pt‐sty‐*m*BDP_2’_.

The decision to begin with complexes Pt‐sty‐3BDP_1_ and Pt‐*m*BDP_2_ is motivated by the primary interest in understanding how the positioning of the platinum complex within the BDP core influences the photophysical properties and reactivity of the characterized Pt‐BDP conjugates [22, 33]. These two complexes differ both in the linkage between the two components, metal complex and chromophore, and in the substitution pattern of the BDP scaffold. In complex Pt‐sty‐3BDP_1_, a styryl linker connects a dpa‐based Pt(II) complex to the 3‐position of the BDP_1_ core, which bears methyl groups at positions 1, 5, and 7, and a methyl benzoyl group at the *meso* position. In complex Pt‐*m*BDP_2_, instead, the platinum fragment is directly linked to the *para* position of the phenyl ring already present in the *meso* position of the BDP scaffold. Moreover, this BDP_2_ core features methyl groups at positions 1 and 7, and *para*‐hydroxystyryl groups at positions 3 and 5, which considerably extend the π‐conjugated system of the dye. The experimental absorption maxima reported for these systems are significantly different, 526 versus 654 nm respectively. This computational investigation aims to determine whether the observed differences arise primarily from the distinct linking site of the platinum fragment to the chromophore or from the presence of electron‐donating groups that extend π‐conjugation on the chromophore itself.

The designs of the new complexes, Pt‐sty‐*m*BDP_2_ and Pt‐sty‐*m*BDP_2’_, draw inspiration from the BDP scaffold of Pt‐*m*BDP_2_ and the extended linker of Pt‐sty‐3BDP_1_, that in this way became even much longer. The styryl linker to the *meso*‐phenyl of BDP is common to both the conjugates. They differ in the substituents at positions 1 and 7: in Pt‐sty‐*m*BDP_2_, these positions bear *para*‐hydroxystyryl groups, while in Pt‐sty‐*m*BDP_2’_ they remain as simple methyl groups. For the sake of simplicity, the BDP chromophore characteristics of each conjugate were named sty‐3BDP_1_, *m*BDP_2_, sty‐*m*BDP_2_, sty‐*m*BDP_2’_, as clearly depicted in Scheme 2.

**FIGURE 1:**
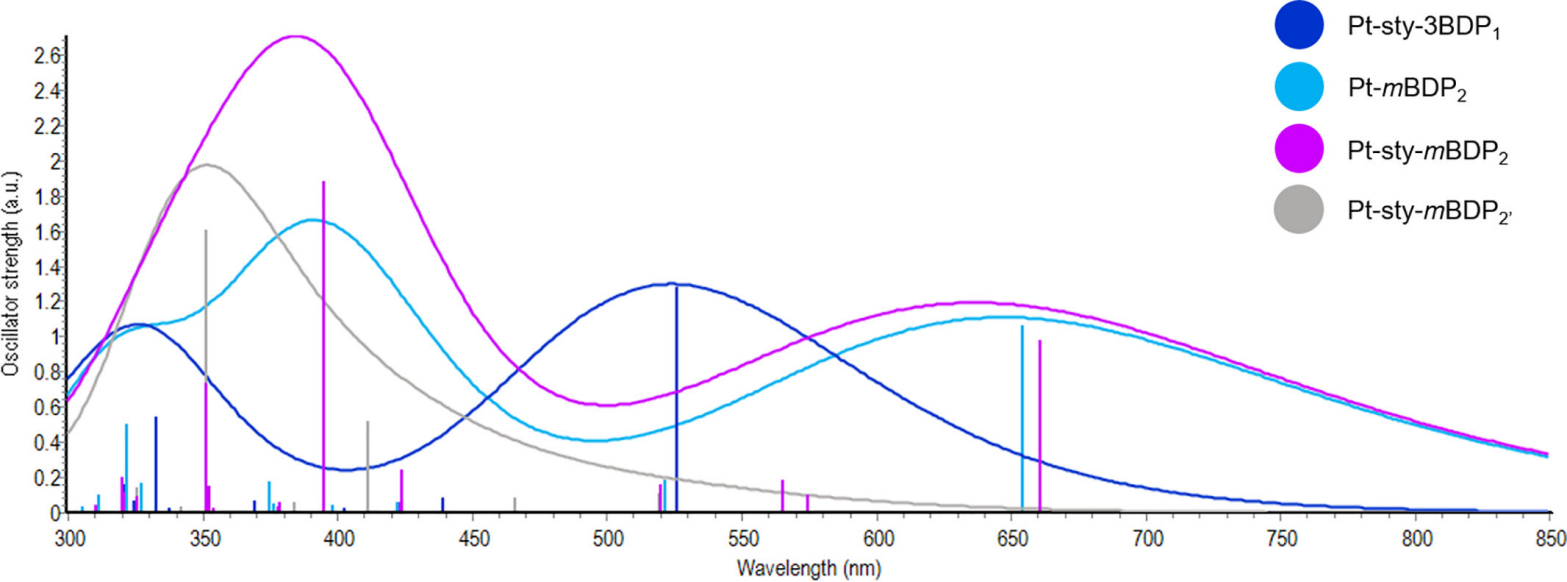
Calculated UV−vis spectra of the conjugates in water solution.

The UV–vis spectra of all the investigated Pt‐BDP conjugates, calculated in a water solution to mimic as much as possible physiological conditions, are presented in Figure 1, while TDDFT details about these spectra and those computed for the BDP chromophores are collected in Tables S1–S4.

All conjugated systems exhibit two intense absorption bands in the UV–visible region, except for the newly formed Pt‐sty‐*m*BDP_2’_, which shows only a single band in the 300–600 nm range, with the maximum absorption wavelength that can be ascribed to a shoulder. While the high‐energy band located in the UV region is less important for therapeutic application, the lowest‐energy visible bands are essential in photoactivated anticancer therapies. A strong absorption in the visible region is typical of BDP dyes, as will be explained in more detail later. To facilitate the comparison between the different conjugates and the BDP chromophore, the maximum absorption wavelength for each studied system is reported in Table 1.

**TABLE 1:** Excitation energies Δ*E* (eV), absorption wavelength *λ* (nm), oscillator strength *f* and MO contributions (%) for the lowest‐energy electronic state of all the investigated systems, conjugates, and BDP chromophore.

Molecular System	Δ*E*	***λ***	***f*** ^**a**^	**MO contribution** ^**b**^	Theoretical assignment
Pt‐sty‐3BDP_1_	2.36	526	1.276	HOMO→LUMO 98%	LC_BDP_
sty‐3BDP_1_	2.36	525	1.092	HOMO→LUMO 97%	ππ*
Pt‐*m*BDP_2_	1.90	654	1.064	HOMO→LUMO 100%	LC_BDP_
*m*BDP2	1.90	653	1.072	HOMO→LUMO 100%	ππ*
Pt‐sty‐*m*BDP_2_	1.88	661	0.976	HOMO→LUMO 97%	LC_BDP_
sty‐*m*BDP_2_	1.88	661	1.001	HOMO→LUMO 99%	ππ*
Pt‐sty‐*m*BDP_2’_	2.39	519	0.111	HOMO‐1→LUMO 99%	LC_BDP_
sty‐*m*BDP_2’_	2.27	547	0.098	HOMO‐1→LUMO 99%	ππ*

The analysis of the natural transition orbitals (NTOs), namely hole and particle, guided the assignment of the band character of each absorption spectrum. The electronic spectrum of the reference complex Pt‐sty‐3BDP_1_ presents two absorption bands, the most intense of which is centered at 526 nm (Table 1), that satisfactorily reproduces the experimental band found at 567 nm [22]. This transition is mainly assigned to a HOMO→LUMO excitation (98%) with ligand‐centered (LC) character. According to the NTOs plot (Figure S1), both hole and particle are localized on the BDP core, including the styryl groups, and no involvement of the metal center occurs, leaving the transition as ππ*. The second, less intense band in the UV region exhibits mixed character, combining intraligand charge transfer (ILCT) within the BDP moiety, metal‐to‐ligand charge transfer (MLCT) involving the tridentate ligand dpa, and finally ILCT associated with charge transfer between the BDP fragment and the dpa directly bound to the metal (Table S1).

The computed UV–vis spectrum of the second reference system, Pt‐*m*BDP_2_, well reproduces the experimental spectrum and displays two main absorption bands [33]. In the 500–850 nm region, a strong absorption peak at 654 nm is observed (Table 1), corresponding to a HOMO→LUMO excitation (100%) with LC character, where both donor and acceptor orbitals are localized on the BDP core, without a significant involvement of the linker, here represented by a phenyl ring (Figure S1). The higher‐energy and most intense band is centered at 395 nm and mainly originates from HOMO → LUMO + 4 (59%) and HOMO‐1 → LUMO (16%) excitations. As for Pt‐sty‐3BDP_1_, this high‐energy band involves transitions with mixed ILCT, MLCT, and LC characters (Table S2).

The simulation of the absorption spectrum of the chromophores, already bearing the proper linker, allows observing that in the case of the synthesized conjugates, Pt‐sty‐3BDP_1_ and Pt‐*m*BDP_2_, the absorption features are entirely due to the chromophore characteristics. The maximum absorption wavelengths of 525 and 653 nm computed for sty‐3BDP_1_ and *m*BDP_2_, respectively, perfectly fit the absorption wavelengths of the corresponding conjugates, whose low‐frequency absorption is peaked at 526 and 654 nm, respectively (Table 1).

**FIGURE 2:**
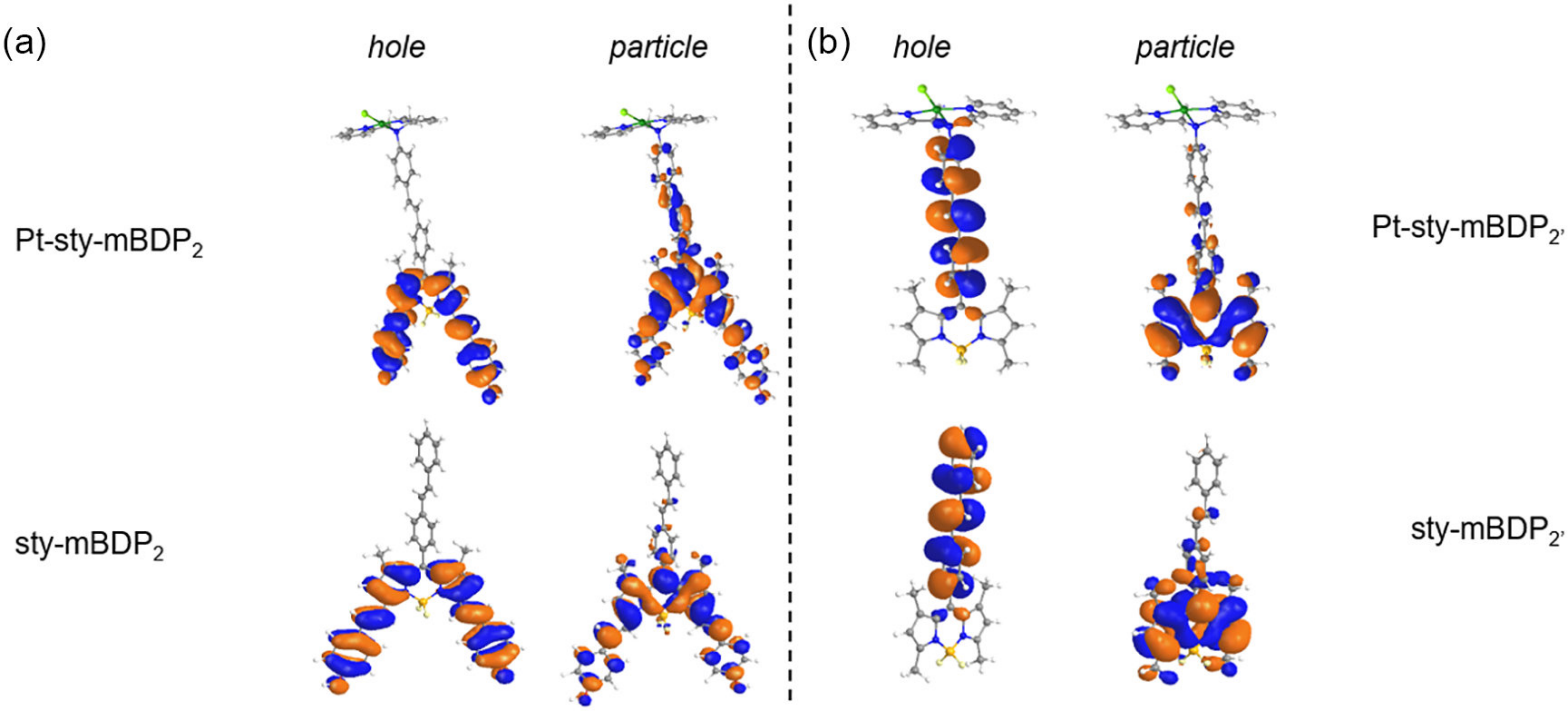
Natural transition orbital plots, hole and particle, for the maximum absorption of the designed conjugates and the corresponding chromophores, panel (a) and (b) for sty‐mBDP_2_ and sty‐*m*BDP_2'_ chromophores, respectively. Orange and blue colors stand for gained and lost electron density, respectively, in hole, the orbital where the electron leaves, to particle, where the electron goes. NTO isosurfaces are generated using the Chemissian software v4.67 and an isovalue of 0.02 a.u.

Similarly to Pt‐*m*BDP_2_, the Pt‐sty‐*m*BDP_2_ conjugate displays a redshift of maximum absorption up to 661 nm, corresponding to the first electronic transition (Table 1). Molecular orbitals analysis indicates that this *λ* ^max^ is dominated by the HOMO → LUMO excitation (97%) with LC character, with both NTOs localized on the BDP core (Figure 2). The second, more intense band is centered at 395 nm, and the overall spectrum closely matches that of Pt‐*m*BDP_2_ (Table S3). The difference between the two conjugates arises from the extended linker, which effectively promotes a redshift of the maximum absorption wavelength. Consistently, the electronic spectrum of the corresponding chromophore, sty‐*m*BDP_2_, exhibits a strong absorption band at essentially the same energy, confirming that the bathochromic shift is primarily driven by the expanded π‐conjugation.

In contrast, Pt‐sty‐*m*BDP_2’_ conjugate exhibits a remarkable different absorption profile compared to the reference conjugate Pt‐*m*BDP_2_ and then the other designed Pt‐sty‐*m*BDP_2_. The structural variation with respect to the latter regards the substitution at the 3 and 7 positions of the BDP core, that in Pt‐sty‐*m*BDP_2’_ are functionalized with methyl groups instead of the hydroxystyryl substituents present in the Pt‐sty‐*m*BDP_2_. Because of the reduced π‐conjugation, the strong visible absorption between 500 and 600 nm disappears, though an electronic transition arising from HOMO‐1→LUMO excitation remains at 519 nm (with low oscillator strength). However, the spectrum shape is dominated by a single band centered at 351 nm, associated with the HOMO‐1→LUMO + 2 transition (89%) with LC character (Table S4). From the comparison between the conjugate and the chromophore, it can be seen this is the only case in which the inclusion of Pt moiety worsens the absorption properties of the conjugate with respect to the chromophore alone. In the case of the conjugate Pt‐sty‐*m*BDP_2’_, although the particle orbital remains essentially identical to that of sty‐*m*BDP_2’_, the hole distribution involves the nitrogen atom of the dpa ligand. This additional contribution appears to be responsible for the observed blue shift of the maximum absorption wavelength (Figure 2b). The NTOs analysis of the Pt‐sty‐*m*BDP_2_ conjugate clearly evidences that the presence of the Pt complex does not have any significant impact on the charge transfer process associated with the excitation (Figure 2a).

Comparison of the two designed complexes indicates that, beyond linker extension, the presence of hydroxystyryl substituents in Pt‐sty‐*m*BDP_2_, further expanding the π‐conjugation of the chromophore, plays a decisive role in modulating its electronic structure and, consequently, its photophysical properties. This structural modification effectively promotes a bathochromic shift of the absorption band toward the red region of the spectrum, which is particularly advantageous for therapeutic applications.

### 2.2 Type II Photoreactions

Photodynamic action via type II photoreactions relies on efficient triplet‐state population upon excitation, as these states enable energy transfer to molecular oxygen, generating cytotoxic singlet oxygen ^1^O_2_. Therefore, a radiationless ISC from the singlet excited state (S_1_) to a nearby triplet state is required to trigger molecular oxygen excitation. In metal complexes, this process is often facilitated by SOC, a relativistic effect that couples orbital angular momentum and spin, thereby mixing electronic states of different multiplicities and enabling otherwise spin‐forbidden transitions.

According to Fermi’s Golden Rule, evaluation of ISC kinetics and determination of the associated rate constant (k_ISC_) require (i) the relative energies of the electronic states involved in the ISC process at their equilibrium geometries, (ii) a detailed characterization of the vibrational wavefunctions to account for vibronic coupling between the states, and (iii) calculation of the SOC matrix elements. The energy gaps between the coupled states were determined by optimizing the geometries of the relevant singlet and triplet excited states. Triplet‐state optimizations were carried out using the unrestricted Kohn–Sham formalism, starting from the corresponding TD‐DFT optimized structures. Vibrational frequency calculations and the associated Hessian matrices were obtained at the optimized excited‐state geometries and employed to derive adiabatic energy differences. SOC matrix elements between the selected states were evaluated as expectation values of the spin–orbit operator within the effective core potential framework. The principal computed parameters, energy gaps, SOC values, and k_ISC_, are reported in Table 2.

**TABLE 2:** Energy splitting Δ*E* (eV) between the lowest singlet state S1 and triplet states lying below at their equilibrium geometry. SOC elements (cm^−1^) calculated at each triplet state optimized structure. Kinetic constant k_ISC_ (s^−1^) for ISC.

	**Pt‐‍sty‐‍3BDP**_**1**_	**Pt‐*‍m*BDP**_**2**_	**Pt‐‍sty‐‍*m*BDP**_**2**_	**Pt‐‍sty‐‍*m*BDP**_**2’**_	
	S_1_–T_1_	S_1_–T_1_	S_1_–T_1_	S_1_–T_1_	S_1_–T_2_
ΔE	0.71	0.53	0.51	0.64	0.26
SOC	0.11	0.12	0.45	0.45	0.39
k_ISC_	1.39·10^5^	4.45·10^5^	1.61·10^7^	2.25·10^6^	6.72·10^6^

TD‐DFT results indicate that all conjugates access a single triplet state located 0.51–0.71 eV below the lowest‐energy singlet excited state (S1) with significant oscillator strength. The only exception is Pt‐sty‐*m*BDP_2’_, for which two triplet states remain lower in energy than S_1_ after optimization of the excited‐state equilibrium geometries.

Spin‐density analysis of the triplet states, performed to clarify their electronic character, reveals that they are all LC states (Figure S2), confirming the dominant role of the BDP core in governing the photophysical behavior. All triplet states show some degree of linker involvement in the spin density: partial in the case of the *meso*‐linked Pt moiety, and more extensive in Pt‐sty‐3BDP_1_, the only conjugate in which the platinum‐containing fragment is attached at a different position on the BDP scaffold. Notably, significant spin‐density contribution from the nitrogen atom of the dpa ligand is observed for T_1_ of Pt‐sty‐3BDP_1_ and T_2_ of Pt‐sty‐*m*BDP_2’_, a feature absent in the other triplet states (Figure S2).

The computed SOC values suggest that ISC enhancement does not originate predominantly from direct Pt‐induced heavy‐atom effects. SOC values on the order of 10^−1^ cm^−1^ are typical of BDP dyes lacking heavy atoms directly attached to the chromophore [34, 35, 36, 37], although fusion or condensation of additional rings onto the BDP core can increase SOC by up to an order of magnitude [34, 38]. Indeed, the triplet states remain largely LC, indicating that excited‐state delocalization and chromophore electronic reorganization play the dominant role in governing ISC efficiency. Only in systems displaying greater electronic integration between the Pt fragment and the BODIPY scaffold is a more noticeable SOC enhancement observed [31]. Consistently, the calculated ISC rate constants fall within the typical range of chromophore‐dominated systems (10^5^–10^7^ s^−1^). Nevertheless, among the investigated conjugates, Pt‐sty‐*m*BDP_2_, the compound exhibiting the most pronounced redshift, also shows the fastest ISC process.

The energy gap between the ground and the lowest excited singlet state of molecular oxygen is a key thermodynamic parameter governing photosensitized singlet oxygen generation. Molecular oxygen in its ground state exists as triplet oxygen, ^3^ *Σ*_g_‐, characterized by two unpaired electrons occupying degenerate π* antibonding orbitals with parallel spins. The lowest excited state, ^1^Δ_g_, corresponds to a spin‐paired configuration of these electrons and lies ≈0.90 eV above the ground state. This energy difference defines the minimum threshold that must be supplied by an excited sensitizer in order to enable triplet–triplet energy transfer and promote the formation of singlet oxygen. For efficient photosensitization, the triplet excited state (*T*_1_) of the conjugates must, therefore, possess an energy equal to or greater than ~0.90 eV. If the *T*_1_ energy is below this threshold, the energy transfer process becomes endergonic, and singlet oxygen generation is thermodynamically unfavorable. Conversely, when the triplet energy exceeds 0.90 eV, energy transfer from the sensitizer to molecular oxygen becomes energetically allowed.

In the present case, the computed triplet energies of all investigated conjugates fall within the range of 1.03–1.57 eV (Figure S2). These values are consistently higher than the required excitation energy of oxygen, providing a positive driving force for the ^3^ *Σ*_g_‐ → ^1^Δ_g_ transition. The energy excess (0.13–0.67 eV above the oxygen threshold) is sufficient to compensate for inevitable non‐radiative losses and vibrational relaxation effects that accompany real photophysical processes.

Therefore, from a thermodynamic standpoint, all conjugates possess triplet states energetic enough to sensitize singlet oxygen formation. While additional factors such as triplet lifetime and oxygen diffusion will influence the overall quantum yield of ^1^O_2_ production, the calculated *T*_1_ energies clearly confirm that the primary energetic requirement for singlet oxygen generation is fulfilled.

From an experimental perspective, the calculated oscillator strengths are consistent with the intense visible absorption expected for efficient photosensitizers, whereas the redshifted absorption observed for Pt‐*m*BDP_2_ and Pt‐sty‐*m*BDP_2_ is particularly advantageous for tissue penetration within the therapeutic window. The localization of spin density mainly on the BODIPY scaffold confirms that the triplet states retain predominantly chromophore‐centered character, supporting efficient energy transfer to molecular oxygen. Furthermore, the computed triplet energies above the 0.90 eV threshold are compatible with experimentally observable singlet oxygen generation through type II photodynamic processes.

### 2.3 Pt‐BDP Conjugates Aquation and DNA Binding

**SCHEME 3:**
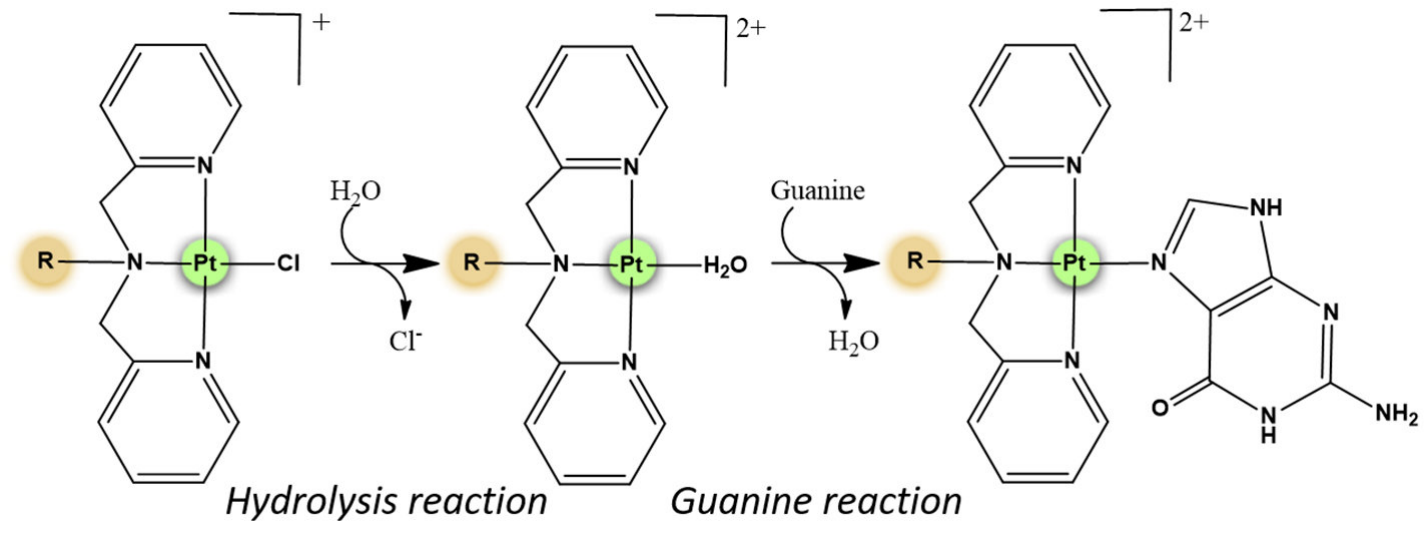
Investigated hydrolysis process for compounds A–D and subsequent guanine reaction of the aquated complexes.

To evaluate the impact of the BDP scaffold on the activation mechanism of the Pt(II) complexes, the hydrolysis process and the DNA‐platination by the conjugates were investigated. Therefore, both the chlorido–water substitution reaction and the subsequent binding to guanine, known as the most probable DNA site attack for the widely used cisplatin‐like compounds, were explored [5]. The reaction pathways examined by quantum mechanical DFT calculations are schematically reported in Scheme 3.

**FIGURE 3:**
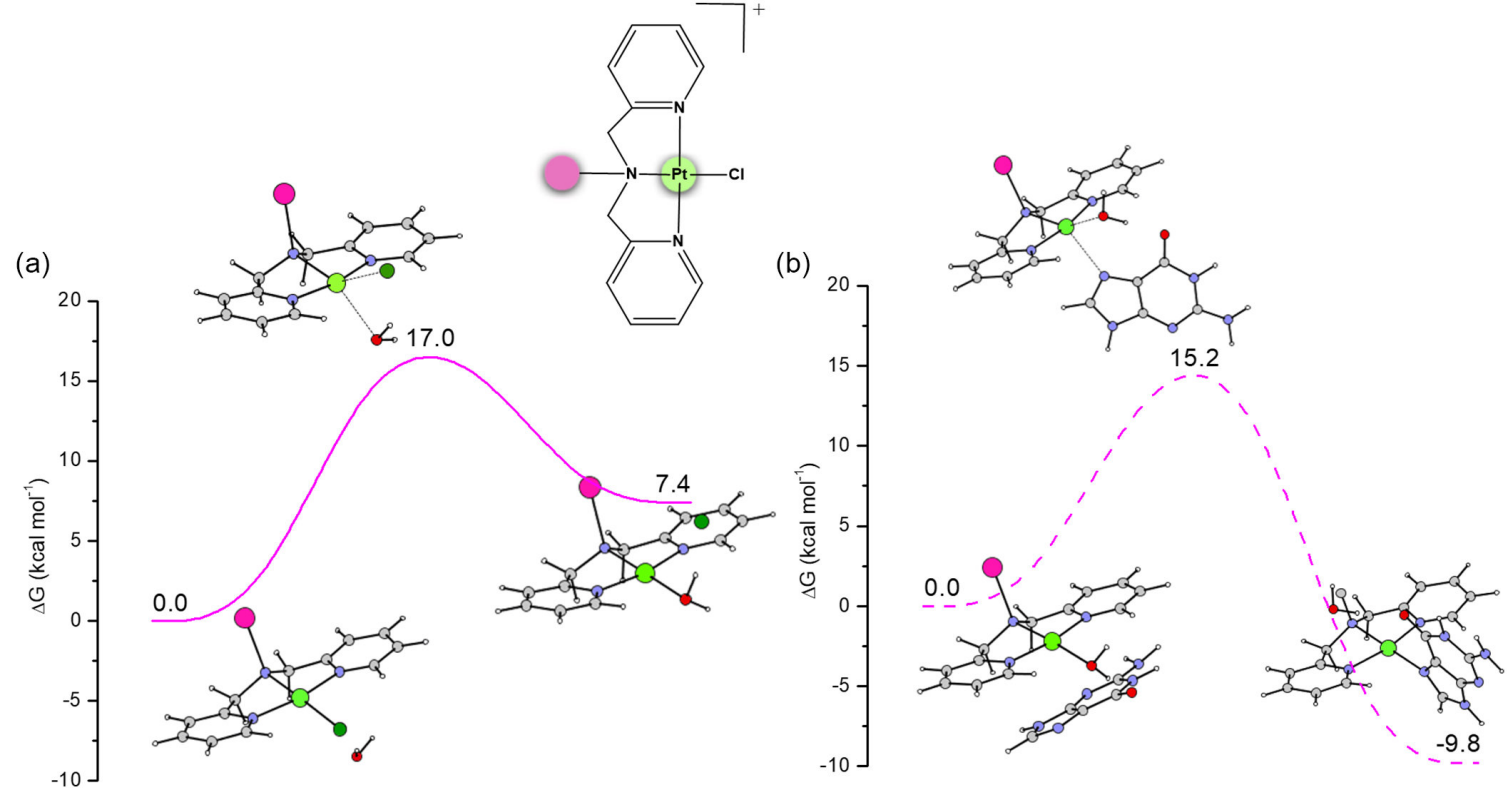
Calculated M06L free energy profiles describing the (a) hydrolysis mechanism of the Pt‐sty‐*m*BDP_2_ conjugate leading to the formation of Pt(II)‐aquated product and (b) water substitution in favor of guanine. Energies are in kcal mol^−1^ calculated with respect to the initial adducts.

**FIGURE 4:**
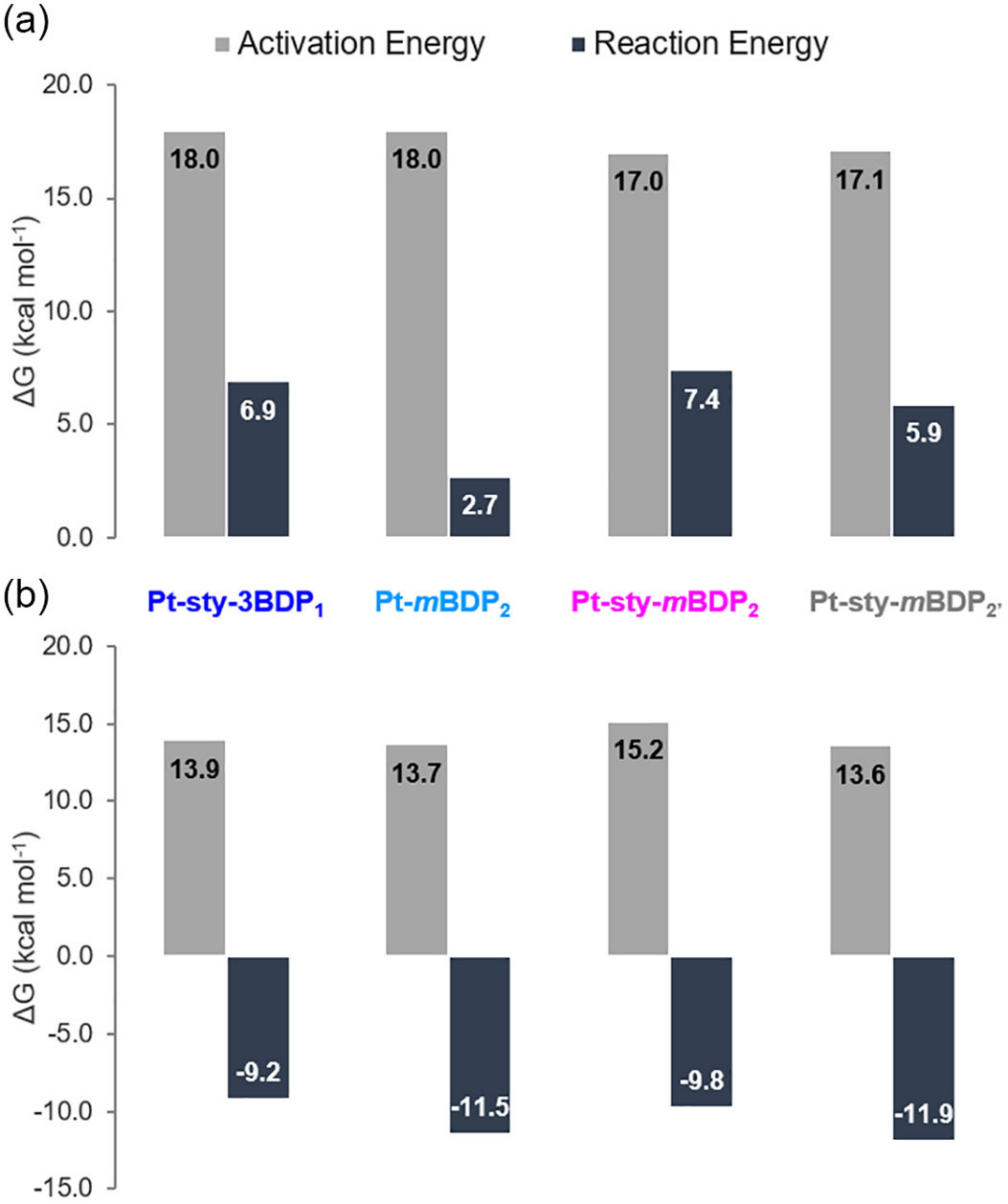
Calculated activation energy barriers and relative energies of the final products in solution (kcal mol^−1^) for the (a) hydrolysis reactions and (b) guanine attack of Pt–BDP conjugates.

It is well known that cisplatin‐like complexes primarily target DNA, forming mono‐ or bifunctional adducts through coordination to the *N*7 position of guanine bases, thereby inhibiting the transcription process. The aquation reaction occurs via a typical interchange mechanism, often referred to as second‐order nucleophilic substitution (S_*N*_2), in which a water molecule replaces the chlorido ligand. For the complexes investigated in this study, as they are monofunctional Pt(II) species, only one step of hydrolysis can occur arising from chlorido ligand substitution. The hydrolysis therefore proceeds via a transition state in which a water molecule approaches the metal center and displaces the chlorido ligand. Here, to better account for the solvation of the chlorido ligand, beside the reactant water molecule, five extra water molecules were added. The potential energy surfaces for the hydrolysis of the conjugate Pt‐sty‐*m*BDP_2_ and guanine coordination to the aqua complex were included in Figure 3 panels a and b, respectively. The activation energy barriers and reaction energies for hydrolyzed products and Pt‐guanine adduct formation for all the conjugates are summarized in Figure 4.

In the initial adduct, the reactant water molecule approaches the Pt(II) center at a distance of ≈4.00 Å in all investigated systems, except for Pt‐sty‐mBDP2’, where it is located closer to the metal center, reaching a distance of 3.28 Å. As expected, in the transition‐state (TS) structure, the Pt─Cl bond is significantly elongated, going from 2.73 to 2.76 Å in all the complexes, except for Pt‐sty‐mBDP_2’_ in which the distance reaches 2.84 Å. The entering water molecule moves closer to the metal center compared to the initial adduct, reaching a distance lower than 2.55 Å in all compounds. The analyses of vibrational modes of TSs clearly show the breaking of the Pt─Cl bond and the simultaneous formation of the Pt─O water bond. The computed relative energies of the transition state are in the range 17–18 kcal mol^−1^. (Figure 4). All the identified transition states are characterized by imaginary frequencies of 121*i*, 119*i*, 131*i*, and 127*i* cm^−1^, confirming their nature as first‐order saddle points (transition states) generally found for interchange mechanisms for the hydrolysis reaction of metal complexes [39, 40, 41, 42, 43, 44].

The formation of the final aqua complexes, in which the chlorido ligand, displaced by the water molecule, remains weakly interacting with the aqua complex, is calculated to be endergonic in all cases. The reaction energies are 6.9, 2.7, 7.4, and 5.9 kcal mol^−1^ while going from Pt‐sty‐3BDP_1_ to Pt‐sty‐mBDP_2’_ complexes. The lowest value was obtained for the Pt‐mBDP_2_ system, in which the water molecules in the second coordination sphere of the metal weakly interact with the BDP fragment, establishing favorable interactions that stabilize the final product. The Pt‐OH_2_ distances are ≈2.11 Å for all the complexes.

The analysis of the hydrolysis process for all investigated systems indicates that the Pt(II) moiety follows an activation mechanism analogous to that of cisplatin and its analogs [5, 21, 29, 30]. Moreover, the theoretical results reveal no significant differences among the computed activation energy barriers, suggesting that the presence of the BDP chromophore has a negligible influence on the aquation step.

Following hydrolysis, the interaction of the aqua complexes with DNA was studied by considering the nucleophilic attack of the *N*7 atom of guanine on the platinum center, accompanied by the displacement of the coordinated water molecule. For all systems, the reaction begins with the formation of adducts in which the guanine base is arranged in a parallel manner with respect to the dpa tridentate ligand. As observed for the aquation process, this step proceeds via a typical interchange mechanism, involving the replacement of the water ligand by a guanine base. In the transition state structures, the Pt–OH_2_ bond distance is ≈2.42 Å, while the forming Pt─*N*7 bond is about 2.53 Å for all the studied complexes. The calculated activation energy barriers are between 13.6 and 15.2 kcal mol^−1^. In all cases, the interchange reactions, leading to the formation of the final products in which the guanine occupies a coordination site of the square planar complex, are exergonic, between 9.2 and 11.9 kcal mol^−1^. The resulting products exhibit Pt–*N*7 bond distances of ≈2.05 Å.

The calculated reaction profiles indicate that the aquation step is slightly endergonic for all investigated complexes. Such behavior is, nevertheless, consistent with the known chemistry of cisplatin‐like Pt(II) compounds, for which hydrolysis is generally reported to be thermoneutral or moderately endergonic depending on the computational approach and solvation treatment. Importantly, aquation is a reversible process strongly influenced by the biological environment, particularly chloride concentration and solvent stabilization effects. Under intracellular conditions, characterized by a significantly lower chloride concentration compared to extracellular media, the equilibrium is shifted toward the aquated species, thereby facilitating subsequent coordination to biological nucleophiles.

In agreement with this interpretation, the subsequent guanine coordination step was found to be kinetically accessible and thermodynamically favorable relative to the aquated intermediate. Therefore, although the overall process is not predicted to be strongly downhill, the calculated energetics remain compatible with the activation mechanism commonly proposed for monofunctional cisplatin‐like complexes.

## 3 Conclusion

This article provides a comprehensive computational investigation of BODIPY‐decorated monofunctional platinum(II) complexes, elucidating the interplay between chromophore design and metal‐centered reactivity in dual chemo‐photodynamic systems.

From a photophysical perspective, the results clearly demonstrate that the optical properties are primarily governed by the BDP scaffold rather than the Pt(II) fragment. Strategic extension of π‐conjugation through styryl linkers and hydroxystyryl substituents proved decisive in achieving a bathochromic shift of the absorption band into the therapeutic window. Among the designed systems, Pt‐sty‐*m*BDP_2_ exhibits the most promising profile, with a calculated *λ* ^max^ at 661 nm and efficient ISC. In contrast, reduced conjugation in Pt‐sty‐*m*BDP_2’_ leads to blueshifted absorption and diminished oscillator strength, highlighting the critical role of chromophore engineering.

Triplet state analysis confirms that all conjugates possess T_1_ energies well above the 0.90 eV threshold required for singlet oxygen sensitization, ensuring thermodynamically favorable energy transfer to molecular oxygen. Although the heavy‐atom effect of platinum only modestly enhances SOC, the calculated ISC rate constants fall within the 10^5^–10^7^ s^−1^ range, consistent with effective type II photoreactivity.

Importantly, the incorporation of the BDP photosensitizer does not compromise the intrinsic reactivity of the platinum center. The computed hydrolysis barriers and subsequent guanine coordination energetics are comparable across all conjugates and align with established cisplatin‐like compounds' activation mechanisms.

Overall, the calculations identify general design principles for Pt–BODIPY conjugates. Extension of π‐conjugation through styryl linkers and hydroxystyryl substituents is the primary factor governing bathochromic shifts and enhanced visible‐light absorption. In contrast, the Pt(II) fragment exerts only a minor direct influence on the electronic excitations unless stronger electronic coupling with the chromophore is established. Efficient triplet‐state population is therefore mainly controlled by chromophore‐centered excited states and by the degree of electronic delocalization within the BODIPY scaffold. These findings provide practical guidelines for the rational design of dual chemo‐photodynamic systems operating within the therapeutic window.

## 4 Computational Details

All the calculations were performed using Gaussian 16 package [45], at the DFT and TD‐DFT levels of theory. The M06L exchange–correlation functional was employed, based on a prior benchmark study carried out on the Pt‐sty‐3BDP_1_ complex. The selection of the M06L functional was based on the performance of a series of exchange‐correlation functionals in reproducing the maximum absorption wavelength (*λ* ^max^) experimentally found, as reported in Table S5 of the Supporting Information section. Solvent effects were accounted for by the SMD implicit solvation model [46], with water represented by a dielectric constant of *ε* = 80. The Pt center was described using the def2 effective core potential (def2‐ECP) [47] and the corresponding valence basis set, while the def2‐SVP basis set [48] was used for all other atoms. The UV–vis spectra were computed within the nonequilibrium time‐dependent TDDFT approach in water solution as vertical electronic excitations from the optimized ground‐state geometries. NTOs plots were generated using the Chemissian software v4.67 [49].

The hydrolysis process and the subsequent reaction between aqua complexes and guanine nucleobase, were explored at the same level of theory. In the former case, explicit microsolvation was included to ensure proper solvation of the leaving chlorido ligand. To this end, six water molecules were introduced around the complex. The nature of all optimized stationary points, minima, and transition states was confirmed by harmonic vibrational frequency analysis. Thermochemical corrections derived from these calculations were used to compute Gibbs free energies. Intrinsic reaction coordinate (IRC) calculations were performed to verify the proper connectivity of each transition state with the corresponding reactant and product minima.

The ISC rate constants between low‐lying singlet and triplet excited states were investigated with ORCA code (version 5.0.4) [50]. The geometries of both the singlet and low‐lying triplet excited states were optimized within the TDDFT framework. Subsequently, the obtained triplet state structures were further optimized within the unrestricted Kohn−Sham (UKS) formalism with the purpose of reducing instability associated with the excited triplet states [51, 52]. This procedure was effectively adopted in our previous work [26, 41, 53]. The Hessian matrices of the involved excited states were computed and the rate constants were obtained as the average of the contributions of the three spin substates (MS) = {+1, 0, −1}. M06L functional was used coupled with the ZORA‐def2‐SVP basis set for all atoms, while the SARC‐ZORA‐SVP basis set was adopted for the Pt center. The RIJCOSX approximation was applied to improve computational efficiency, following the recommendations provided in the ORCA manual.

## Conflicts of Interest

The authors declare no conflicts of interest.

## Supporting information

Supplementary Material

## Data Availability

The data that support the findings of this study are available from the corresponding author upon reasonable request.
