# Supplementary material for: Computational Analysis of Chromophore‐Controlled Photoactivation in Monofunctional Platinum(II)–BODIPY Conjugates for Dual Chemo‐Photodynamic Therapy
Source: Chembiochem. 2026 Jun 11;27(11):e70422. doi: 10.1002/cbic.70422 (PMC13261104; doi:10.1002/cbic.70422)
Supplement: Supplementary file 1 — Supplementary Material [file CBIC-27-e70422-s001.pdf]

Supporting Information

Computational Analysis of Chromophore-Controlled Photoactivation in Monofunctional Platinum(II)–BODIPY Conjugates for Dual Chemo-Photodynamic Therapy

Fortuna Ponte,*[a] and Gloria Mazzone*[a]

[a] Department of Chemistry and Chemical Technologies, University of Calabria, Via P. Bucci, 87036 Rende (CS) - Italy
E-mail: fortuna.ponte@unical.it, gloria.mazzone@unical.it

Table of Contents

Table S1: Excitation energies ΔE (eV), absorption wavelength λ (nm), oscillator strength f and MO contribution (%) for selected transitions of the chromophore sty-3BDP$_1$ and the corresponding conjugate Pt-sty-3BDP$_1$

Table S2: Excitation energies ΔE (eV), absorption wavelength λ (nm), oscillator strength f and MO contribution (%) for selected transitions of the chromophore *m*BDP$_2$ and the corresponding conjugate Pt-*m*BDP$_2$

Table S3: Excitation energies ΔE (eV), absorption wavelength λ (nm), oscillator strength f and MO contribution (%) for selected transitions of the chromophore sty-*m*BDP$_2$ and the corresponding conjugate Pt-sty-*m*BDP$_2$

Table S4: Excitation energies ΔE (eV), absorption wavelength λ (nm), oscillator strength f and MO contribution (%) for selected transitions sty-*m*BDP$_{2'}$ and the corresponding conjugate Pt-sty-*m*BDP$_{2'}$

Figure S1: Natural Transition Orbitals (NTOs) for the S1 state of the Pt-sty-3BDP$_1$, and Pt-*m*BDP$_2$.

Figure S2: Spin density distributions of the optimised structures of excited triplet states of Pt-sty-3BDP$_1$, Pt-*m*BDP$_2$, Pt-sty-*m*BDP$_2$ and Pt-sty-*m*BDP$_{2'}$ at the TD-DFT M06L/ def2-ECP/def2SVP level of theory.

Table S5. TD-DFT benchmark for the reproduction of the experimental spectrum of the Pt-sty-3BDP$_1$ complex on the structure optimized at M06L/def2-ECP/def2-SVP level of theory in implicit water solvent.

Table S1

	Band	ΔE	λ	*f* [a]	MO contribution [b]	Theoretical assignment
Sty-3BDP$_1$	I	2.36	*525*	1.092	H→L 97%	π- π*
		2.77	*447*	0.106	H→L+1 97%	
	II	3.41	*364*	0.059	H–5→L+1 95%	
		3.71	*334*	0.264	H→L+3 57%, H→L+2 20%	
		3.78	*329*	0.247	H→L+2 24%, H→L+3 40%	
		4.27	*291*	0.327	H–8→L 69%	
		4.57	*271*	0.067	H–3→L+2 37%, H–2→L+2 33%	
Pt-sty-3BDP$_1$	I	2.36	*526*	1.276	H→L 98%	LC_{BDP}
		2.82	*439*	0.082	H→L+3 95%	LC_{BDP}
	II	3.36	*369*	0.070	H–8→L 90%	LC_{BDP}
		3.73	*332*	0.547	H→L+5 31%, H–6→L 21%	LC_{BDP}
		3.82	*325*	0.066	H–2→L+4 49%, H–6→L+1 20%	$ML_{Ndpa}CT$
		3.86	*321*	0.061	H→L+8 42%, H–12→L 21%	$ILCT_{BDP,Ndpa}$
		3.87	*320*	0.161	H–2→L+4 38%, H–6→L+1 20%, H–5→L+1 20%	$ML_{Ndpa}CT$

[a] Only vertical transitions with oscillator strength greater than 0.050 are reported. b Only contributions larger than 15% are reported.

Table S2

	Band	ΔE	λ	*f* [a]	MO contribution [b]	Theoretical assignment
*m*BDP$_2$	I	1.90	653	*1.072*	H→L 100%	π- π*
		2.38	521	*0.177*	H–1→L 77%, H→L+1 22%	
		2.94	422	*0.061*	H–2→L 71%, H→L+2 27%,	
	II	3.14	395	*1.244*	H→L+1 60%, H–1→L 16%	
		3.19	389	*0.182*	H→L+1 60%,	
		3.29	377	*0.061*	H–4→L 86%	
		3.86	321	*0.466*	H–9→L 76%	
		3.97	313	*0.106*	H–1→L+2 66%, H–2→L+1 31 %	
Pt-*m*BDP$_2$	I	1.90	654	*1.064*	H→L 100%	LC_{BDP}
		2.38	522	*0.184*	H–1→L 77%, H→L+4 22%	LC_{BDP}
	II	2.94	422	*0.055*	H–4→L 60%, H→L+7 22%	LC_{BDP}
		3.14	395	*1.276*	H →L+4 59%, H–1→L 16%	LC_{BDP}
		3.31	375	*0.173*	H→L+9 62%, H–8→L 18%	$ILCT_{BDP,Ndpa}$
		3.79	327	*0.166*	H–7→L+1 74%, H–3→L+3 19%	$ML_{Ndpa}CT$
		3.85	322	*0.084*	H–3→L+3 51%, H–5→L+2 29%	$ML_{Ndpa}CT$
		3.86	321	*0.505*	H–12→L 67%	LC_{BDP}

[a] Only vertical transitions with oscillator strength greater than 0.050 are reported. b Only contributions larger than 15% are reported

Table S3

	Band	ΔE	λ	f [a]	MO contribution [b]	Theoretical assignment
sty-*m*BDP_2	I	1.88	661	*1.001*	H→L 99%	π- π*
		2.07	559	*0.178*	H–1→L 92%	
		2.31	536	*0.068*	H→L+1 98%	
		2.38	521	*0.178*	H–2→L 74%, H→L+1 20%	
		2.93	423	*0.058*	H–3→L 73%, H→L+3 24%,	
	II	3.03	408	*0.917*	H→L+2 33%, H–2→L+1 30%, H–1→L+1 26%	
		3.19	389	*1.027*	H–2→L+1 42%, H→L+2 37%	
		3.26	380	*0.059*	H–5→L 96%	
		3.52	352	*0.322*	H–2→L 67%	
		3.62	343	*0.446*	H–11→L 19%	
		3.78	328	*0.346*	H–11→L+3 81%	
		3.99	311	*0.059*	H–2→L+3 64%, H–3→L+2 29%	
		4.68	265	*0.078*	H–7→L+1 45%, H–4→L+4 21%	
**Pt-sty-*m*BDP_2**	I	1.88	661	*0.976*	H→L 97%	LC_{BDP}
		2.16	575	*0.103*	H→L+2 97%	LC_{BDP}
		2.19	566	*0.183*	H–1→L 64%, H–2→L 35%	LC_{BDP}
		2.39	520	*0.157*	H–2→L 55%, H–1→L 24%, H→L+5 20%	LC_{BDP}
	II	2.92	424	*0.243*	H–1→L+2 66%, H–2→L+2 21%	LC_{BDP}
		2.93	423	*0.062*	H–5→L 71%, H→L+8 24%	LC_{BDP}
		3.14	395	*1.881*	H→L+5 59%	LC_{BDP}
		3.27	379	*0.060*	H–9→L 97%	LC_{BDP}
		3.52	352	*0.154*	H→L+10 85%	LC_{BDP}
		3.53	351	*0.734*	H–2→L+2 41%, H–1→L+2 19%	$ML_{Ndpa}CT$
		3.81	326	*0.081*	H–8→L+2 64%, H–8→L+1 23%	$ML_{Ndpa}CT/ILCT_{BDP,Ndpa}$
		3.87	321	*0.117*	H–1→L+8 44%, H–15→L 18%	$ML_{Ndpa}CT$
		3.87	320	*0.199*	H–1→L+8 44%, H–15→L 18%	LC_{BDP}

[a] Only vertical transitions with oscillator strength greater than 0.050 are reported. b Only contributions larger than 15% are reported

Table S4

	Band	ΔE	λ	*f* [a]	MO contribution [b]	Theoretical assignment
sty-*m*BDP$_{2'}$	I	2.27	547	*0.098*	H‒1→L	π- π*
		2.77	447	*0.196*	H→L 50%, H→L+1 50%	
		3.06	406	*0.408*	H→L+1 48%, H→L 47%	
		3.24	383	*0.065*	H‒2→L 91%	
		3.60	345	*1.368*	H‒2→L 91%	
		3.75	331	*0.079*	H‒6→L 82%,	
		4.58	271	*0.051*	H‒6→L+1 33%, H‒7→L 28%, H‒1→L+4 24%	
		4.71	263	*0.050*	H‒1→L+3 55%, H‒4→L+1 28%	
**Pt-sty-*m*BDP$_{2'}$**	I	2.39	519	*0.111*	H‒1→L 99%	LC_{BDP}
		2.66	466	*0.085*	H→L+2 69%, H→L 31%	LC_{BDP}
		3.01	412	*0.517*	H→L 65%, H→L+2 30%	LC_{BDP}
		3.23	384	*0.056*	H‒5→L 92%	LC_{BDP}
		3.53	351	*1.602*	H‒1→L+2 89%	LC_{BDP}
		3.81	326	*0.145*	H‒6→L+1 65%, H‒3→L+4 30%	$ML_{Ndpa}CT$
		3.87	321	*0.119*	H‒10→L 74%	$ML_{Ndpa}CT$

[a] Only vertical transitions with oscillator strength greater than 0.050 are reported. b Only contributions larger than 15% are reported

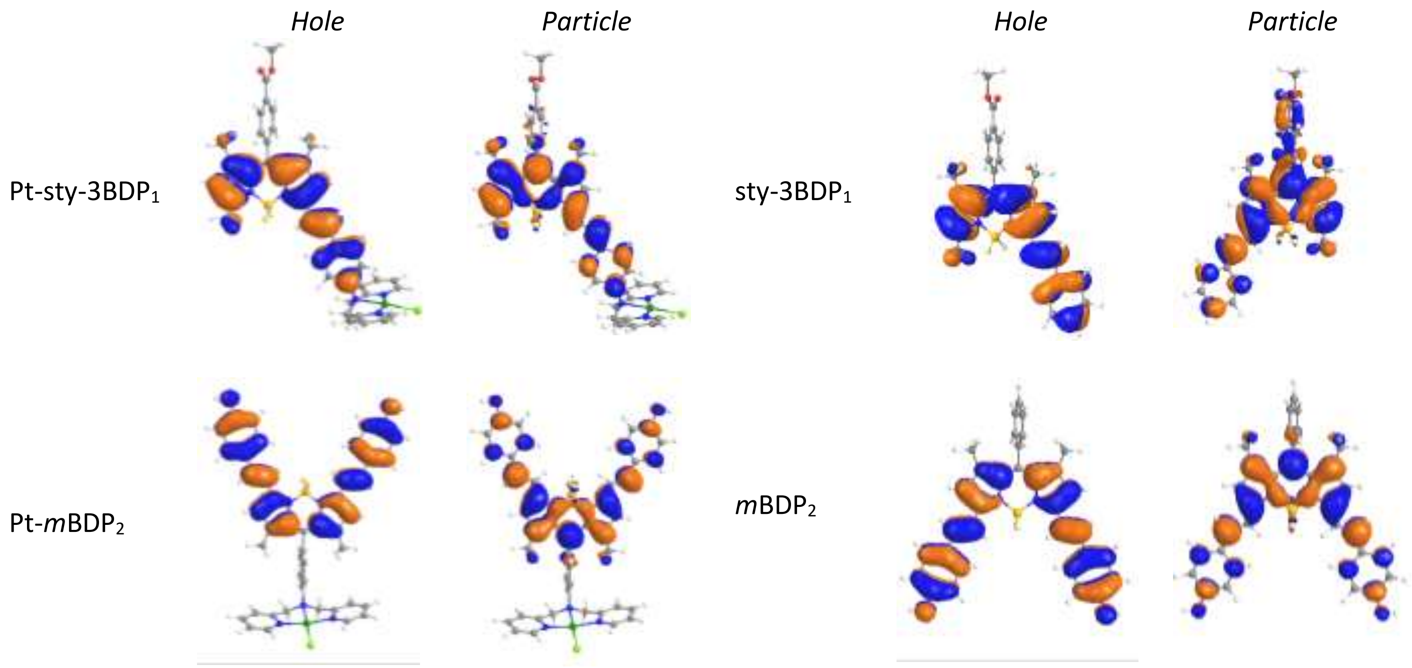

Hole
Particle
Hole
Particle
Pt-sty-3BDP$_1$
sty-3BDP$_1$
Pt-*m*BDP$_2$
*m*BDP$_2$

Figure S1

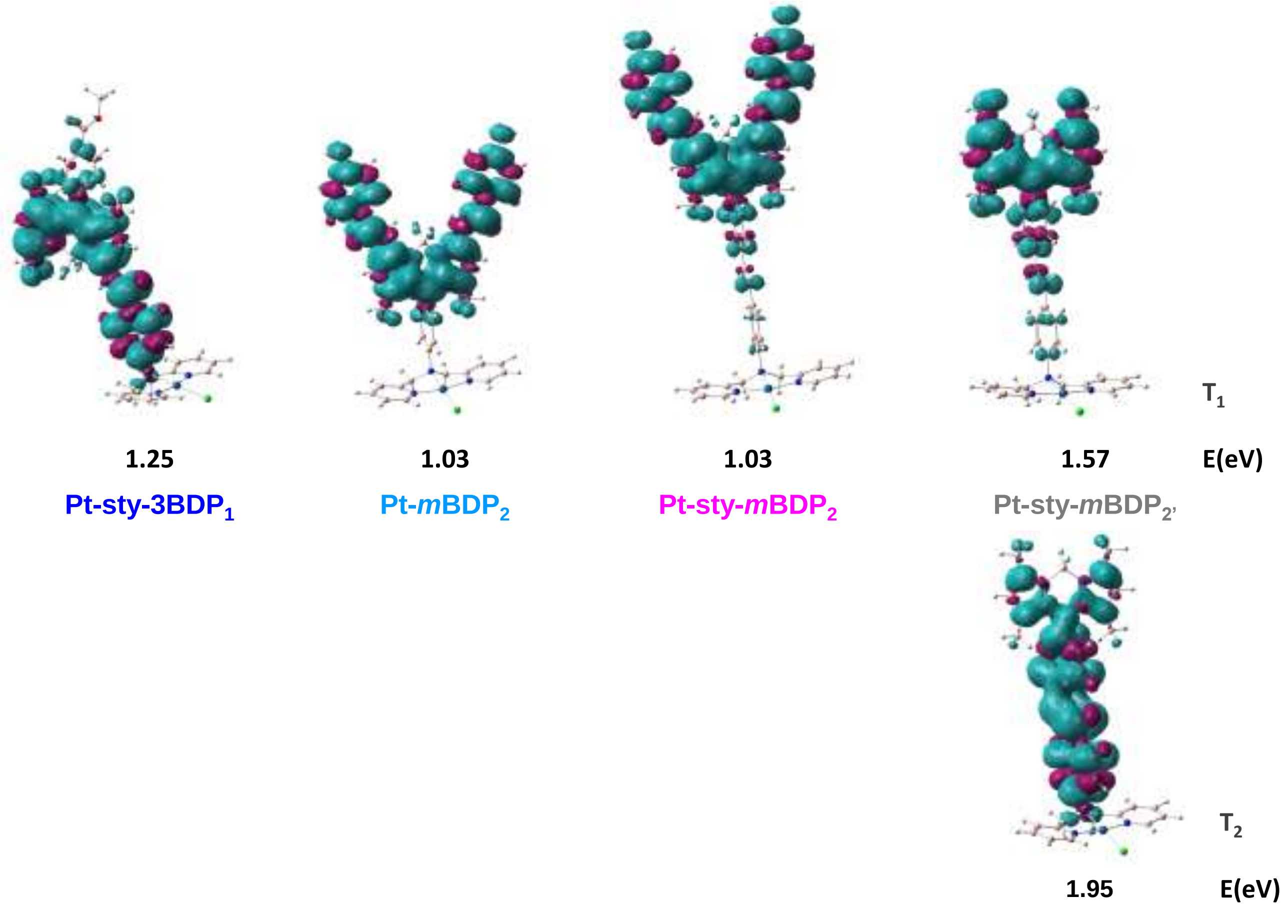
T1
1.25
1.03
1.03
1.57
E(eV)
Pt-sty-3BDP1
Pt-mBDP2
Pt-sty-mBDP2
Pt-sty-mBDP2'
T2
1.95
E(eV)

Figure S2

Table S5

**Functional**	**λ^{max}**	*eV*	*f*	**λ_{exp} [a]**
B3LYP	520	2.39	1.243	567
cam-B3LYP	499	2.48	1.186	
M05	520	2.38	1.232	
M06	522	2.38	1.232	
M06L	526	2.36	1.276	
M062X	501	2.48	1.174	
MN15	512	2.42	1.719	
wB97XD	496	2.50	1.178	

a. data from Angew. Chem. Int. Ed. 2019, 58, 12661 – 12666
